# Computational developments in microRNA-regulated protein-protein interactions

**DOI:** 10.1186/1752-0509-8-14

**Published:** 2014-02-10

**Authors:** Wei Zhu, Yi-Ping Phoebe Chen

**Affiliations:** 1 Department of Computer Science and Computer Engineering, La Trobe University, Melbourne, Australia

**Keywords:** miRNA, Regulation, Protein-protein interaction

## Abstract

Protein-protein interaction (PPI) is one of the most important functional components of a living cell. Recently, researchers have been interested in investigating the correlation between PPI and microRNA, which has been found to be a regulator at the post-transcriptional level. Studies on miRNA-regulated PPI networks will not only facilitate an understanding of the fine tuning role that miRNAs play in PPI networks, but will also provide potential candidates for tumor diagnosis. This review describes basic studies on the miRNA-regulated PPI network in the way of bioinformatics which includes constructing a miRNA-target protein network, describing the features of miRNA-regulated PPI networks and overviewing previous findings based on analysing miRNA-regulated PPI network features.

## Review

### Brief introduction to MicroRNA and protein-protein interaction networks

MicroRNAs (miRNAs) are a subset of small (~22 nucleotide – structural units of RNA and DNA - in length) non-coding RNA molecules, which comprise 1% of genes in animal genomes. The miRNAs are mainly found in intergenic regions (IG region), although some are also located in intronic regions [1,2]. miRNAs can repress gene expression by interacting with three prime untranslated regions (3′ UTR) which are sequences that are not translated into proteins in the 3′end of the target mRNAs. Since Rosalind Lee and Rhonda Feinbaum discovered the first two precursors (pre-miRNAs) - lin-4 and let-7, products of C. elegans genes, miRNAs have been keenly studied for several years [1-6]. miRNAs play an important role in controlling nematode developmental timing and repressing mRNA translation [3,4] at the post-transcriptional level in a gene regulatory network (the gene regulatory network can be divided into the transcription level and post-transcription level [7], during which transcription factors (TF) and miRNAs contribute the most, respectively). Research has also indicated that miRNAs have a close relationship with tumorigenesis [8-10]. Recent research showed evidence that miRNAs can affect cancer initiation and progression [11,12].

MiRNAs have a complete complementarity region (2-8nt) which is called the “seed sequence” in their 5′ end. Most miRNAs are highly conserved among species [13,14]. In RNA regulation, the 5′ region and coding region play important roles in binding to mRNA targets. Thus, a many-to-many relationship structure can be established in miRNAs and their target mRNAs, that is: a miRNA can regulate more than one target mRNA and each target mRNA also can be regulated by more than one miRNA[15]. miRNAs have the ability to post-transcriptional degrade their mRNA target or inhibit translation. Both mechanisms fine-tune mRNA expression by distinguishing sequence motifs in the 3′UTR of mRNA, for example, miR-182 binds with its direct targets, fibroblast growth factor 9 (FGF9) and neurotrimin (NTM) in response to injury stimuli [16]. The length of 3′ UTR indicates the density of miRNA binding sites. It has been shown that the mRNA with a longer 3′ UTR always participates in more sophisticated functions, whereas mRNAs with a shorter length 3′ UTR are generally involved in basic biological processes [17]. Some special mRNAs are regulated by miRNAs in their five prime untranslated regions (5′ UTR). Ørom *et al*. found that miR-10a binds 5′UTR of ribosomal protein mRNAs to enhance their translation [18]. Recent studies show that miRNAs can also bind with their targets in coding regions [19].

The interaction between miRNA and mRNA provides a new way to determine gene functions. In studies on miRNA-mRNA interaction, how to accurately find the target mRNA genes for miRNAs is the most important question. Various approaches have emerged since the discovery of miRNAs. Targeting mRNA genes in plants can easily be done because most miRNAs have perfect and stable complementarities [20]. miRNAs in animals, however, are allowed to have mismatches and gaps in the functional duplex, which generates uncertainty in the target site prediction in miRNAs [21]. Efficient target prediction approaches are discussed later in this paper.

In recent years, researchers have studied miRNA-regulated networks, including miRNA co-regulated networks, miRNA-mRNA networks, miRNA-transcription factor networks and miRNA-protein interaction networks. Currently, research on miRNA-regulated protein-protein interaction networks is in its initial stage. Barriers to this research not only exist because of the mystery of miRNAs, but also due to the complexity of protein-protein interactions.

Protein-Protein Interaction (PPI) is one of the most important tasks required for a living cell to carry out its biological functions such as DNA replication, transcription, translation, signal transduction [22]. PPI can be simulated as an undirected static network structure. The properties of the PPI network can be denoted by a series of elements, edges, nodes, cluster coefficients and so on [23]. The topological features are discussed later in this paper.

The research on PPI networks has developed rapidly since the yeast two-hybrid system was first described in Field and Song’s publication [24]. Based on Y2H, Schwikowski, Uetz and Field released a PPI network map in yeast [25] in 2000, then a human PPI network map was reported in 2005 [26]. Before this, PPI network research generally centred on other species such as yeast- *Saccharomyces cerevisiae*, and worms- *C. elegans*. Currently, proteome-wide studies of PPI networks in different species mostly concentrate on PPI network detection and prediction [24,27-30], signal transduction pathways [31-33], protein function prediction based on PPI networks and protein complex prediction in PPI networks [34-37].

Currently, the research on miRNA-regulated PPI networks can be divided into two main areas: a) basic studies on the correlation between miRNAs and general PPI networks. This subject mainly uses bioinformatics approaches and statistical means to explore the relationship between miRNA or cluster miRNAs and PPI network topological properties linked by miRNA target genes, which aims to find rules in miRNA-regulated gene expression beside seed matching. Although basic studies on miRNA-regulated PPI networks always suffer from partial coverage and false positives and negatives, it is undeniable that the studies are important. The basic studies of miRNA regulation in PPI networks attempt to discover new correlations and tendencies between miRNAs and their target proteins in a relatively fast way, which provides part of the theoretical support to laboratory experiments; and b) identification of the impact of miRNA regulation on PPI networks in diseases. As an essential component of PPI networks and prime candidates of miRNA targeting modulators in animal cells [38], signalling transduction pathways have been extensively explored in recent years. The correlation between miRNAs and signalling pathways was mostly proved by biochemical analysis. For example, Rogler *et al*. [31] found that through down-regulating the target gene Smads (including Smad3, 4 and 5), which are the key genes in transforming growth factor-beta signalling of miR-23b cluster miRNAs (including miR-23b, miR-27b, miR-24-1, miR-10a, miR-26a, and miR-30a), miR-23b miRNAs can be promoted in growth and consequently repress bile duct gene expression in fetal hepatocytes. Jie *et al.*[32] pointed out that miR-146a expression induced by epidermal growth factor receptor (EGFR) signalling can repress human gliomagenesis by down-regulating its target gene, NOTCH1. Recent research [33] shows that miR-128 can play a similar role by targeting oncogenic receptor tyrosine kinases signalling. In this review, we describe the current computational developments in basic studies on the correlation between miRNAs and general PPI networks, which may be helpful to gain new insights into current miRNA-regulated PPI network studies.

### Understanding the role of miRNA in regulating target genes and proteins

**Figure 1:**
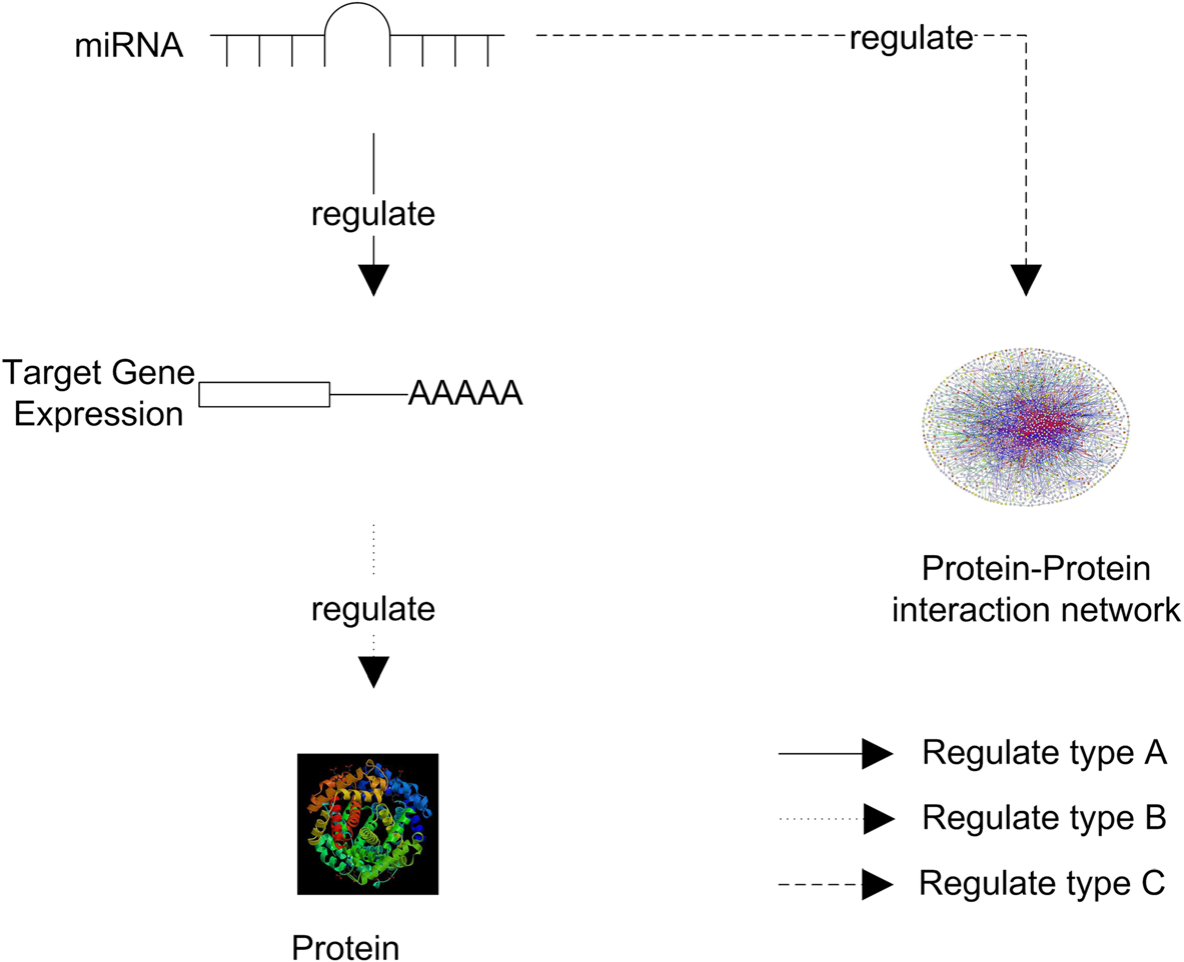
**Types of miRNA regulation.** Type A: MiRNA regulating gene expression. miRNAs can lead to mRNA cleavage and degradation or mRNA translational repression. Type B: MiRNA regulating target protein. The action of under or over expression of miRNAs can directly repress gene translation. Type C: MiRNA regulating on PPI networks. miRNA shows as an indirect regulator to affect dynamic PPI network stability.

MiRNAs are involved in fine-tuning gene expression and biological processes at the post-transcription level by regulating their targets, such as signalling proteins, enzymes, transcription factors (TFs) [39]. For the mechanism of miRNA regulation, it has been accepted that in mature miRNAs directly associated with AGO_1_ (a member of the Argonaute protein family, the main component of RNA-induced silencing complex) in the RNA-induced silencing complex (RISC), the process of target selection occurs in RISC by miRNA strand unwinding, which leads to the repression of mRNA translation, stability and localization [38,40,41]. However, how miRNAs behave in RISC or how miRNAs play a regulation role is still unknown. As shown in Figure 1, we summarized the possible types how miRNAs regulation in PPI network from previous publications. In terms of regulating proteins, miRNAs regulate protein-coding targets to affect protein synthesis. miRNAs are reported as a regulator to fine-tune their target genes. The action of under or over expression of miRNAs can directly repress gene translation [42], and according to [43], miRNAs regulate more than 60% of genes which are related to protein synthesis, although this kind of down-regulation impacts most of their targets by no more than 50% [38,43]. In terms of regulating the PPI network, miRNAs may serve as a regulator by maintaining the stablity of PPI networks [44]. It has been reported that protein-protein interactions express a dynamic state rather than maintaining a static state to keep stability in the external environment [44,45]. Protein abundance, which can be tuned by miRNAs indirectly, is one of the most effective factors in the dynamic robustness of the PPI network [42]. In this case, it can be concluded that miRNAs act as an indirect regulator in PPI network stability. Furthermore, the most important sub-graph of the PPI network is the signalling pathway, hence investigation into the relationship between miRNAs and PPI networks could facilitate a deeper understanding of miRNA-regulated signalling pathways.

### Resources to construct a reliable miRNA-protein network

**Figure 2:**
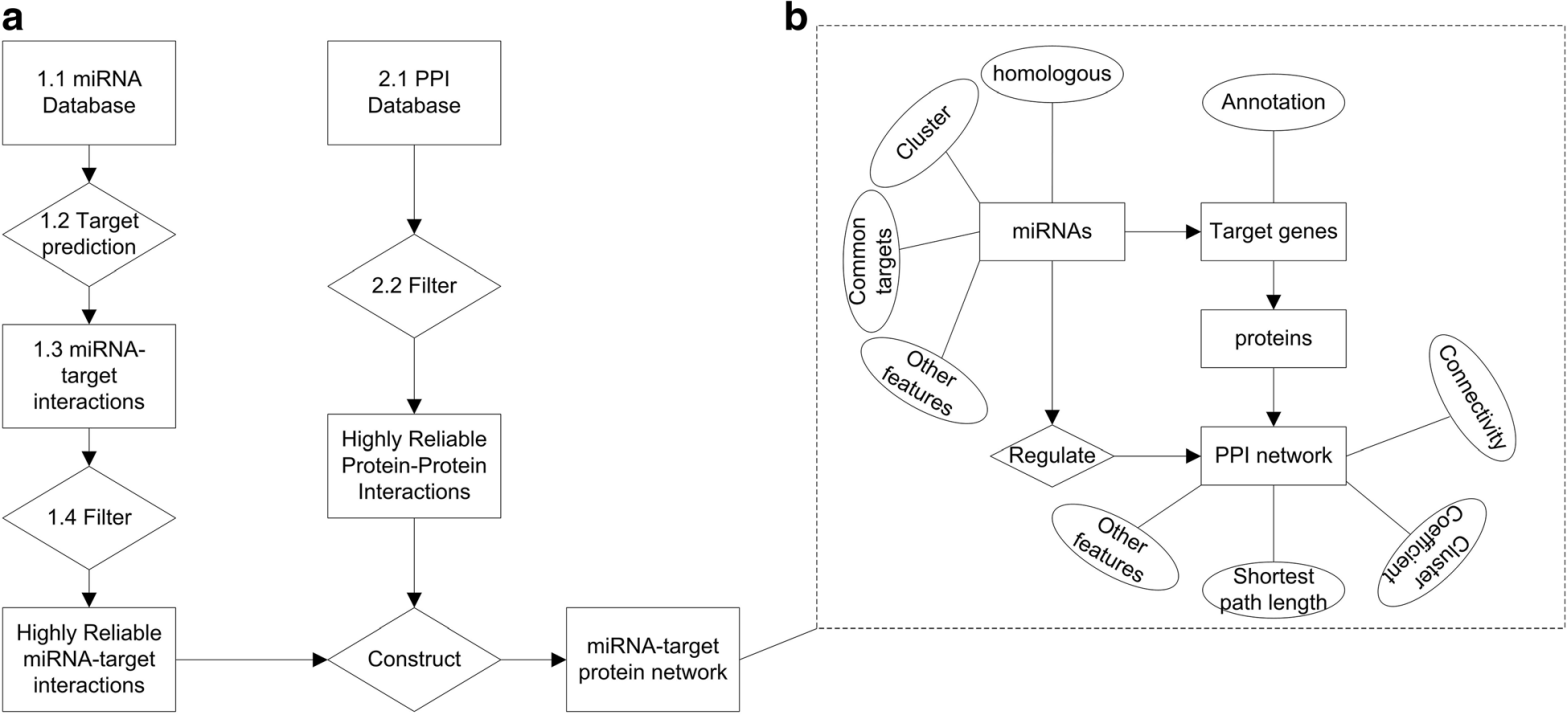
**Constructing a miRNA-target protein network. a)** Process to construct a miRNA-target protein network: the process labels correspond to Table 1 - column 2. **b)** Relationships within miRNA-target protein network and its features.

The construction of a miRNA-protein network (Figure 2a) using highly reliable resources is important for the commencement of a miRNA-regulated PPI network study. As shown in Figure 2, a miRNA–target protein network is constructed by miRNA-target interactions and PPI network data. During the process, the selection of miRNA target predicting approaches and filtering approaches is important to obtain highly reliable data. According to Figure 2a, we divide the resources into the miRNA database (detecting miRNAs based on High Throughput Sequencing (HTS) and miRNA expression profiles included), miRNA target predicting approaches, miRNA-targets interaction database, miRNA-targets interaction filtering tools, PPI database (integrated PPI databases included) and filtering tools for the PPI database, as shown in Table 1. To complete the resources, we also mention platform resources in Table 1. In this section, we introduce several of the tools.

**Table 1:** Resources to construct a reliable miRNA-target protein network

**Name**	**Process**	**Main feature**	**URL**	**Ref.**
BioGrid	2.1	Protein-protein interaction database	http://thebiogrid.org/	[46]
Cytoscape	Platform	Social or molecular networks analysis and visualization.	http://www.cytoscape.org/	[47]
Database of Interacting Proteins (DIP)	2.1	Protein-protein interaction	http://dip.doe-mbi.ucla.edu/dip/	[48]
Ingenuity system	Platform	Signalling and metabolic pathways analysis; molecular network analysis etc.	http://www.ingenuity.com/	
Human Protein Reference Database (HPRD)	2.1	Protein-protein interaction	http://www.hprd.org/	[49]
String	2.1	Protein-protein interaction	http://string-db.org/	[50]
The MIPS mammalian protein-protein interaction database	2.1	protein-protein interaction	http://mips.helmholtz-muenchen.de/proj/ppi/	[51]
Protein Interaction Network Analysis (PINA)	2.2	PPI network construction, filtering, analysis, visualization and management	http://cbg.garvan.unsw.edu.au/pina/	[52]
HitPredict	2.1	Integrated PPI database	http://hintdb.hgc.jp/htp/	[53]
iRefIndex	2.1	Integrated PPI database	http://www.irefindex.org/wiki/index.php?title=iRefIndex	[54]
SynechoNET	1.1	Integrated PPI database	http://bioportal.kobic.re.kr/SynechoNET/	[55]
PMRD	1.1	Plant miRNA database	http://bioinformatics.cau.edu.cn/PMRD/	[56]
Gene ontology	Platform	Gene annotation, develop controlled vocabulary of genes	http://www.geneontology.org/	[57]
MiRTarBase	Platform	miRNA-target interactions	http://mirtarbase.mbc.nctu.edu.tw/	[58]
PicTar	1.2	miRNA target prediction	http://pictar.mdc-berlin.de/	[59]
RNAhybrid	1.2	miRNA target prediction	http://bibiserv.techfak.uni-bielefeld.de/rnahybrid/	[60]
TargetScan	1.2	miRNA target prediction	http://www.targetscan.org/	[61]
GeneSet-2miRNA	1.2	miRNA target predicting with mRNA expression profile	http://mips.helmholtz-muenchen.de/proj/gene2mir/	[62]
MMIA	1.2	miRNA target predicting with mRNA expression profile	http://129.79.244.122/~MMIA/	[63]
miRanda	1.2	miRNA target predicting & miRNA expression profiles	http://www.microrna.org/	[64]
MiRTif	1.4	miRNA target interaction filter	http://mirtif.bii.a-star.edu.sg/	[65]
miRBase	1.1	miRNA sequences and annotations	http://www.mirbase.org/	[66]
The human microRNA disease database (HMDD)	1.1	miRNA sequences and annotations	http://202.38.126.151/hmdd/mirna/md/	[49]
miRExpress	1.1	Extract miRNA expression profiles based on HTS results	http://mirexpress.mbc.nctu.edu.tw/	[67]
TarBase	1.2	Experimental supported miRNA target	http://diana.cslab.ece.ntua.gr/tarbase/	[68]
miRDeep	1.1	Detect novel miRNA based on HTS	https://www.mdc-berlin.de/690237/en/research/research_teams/systems_biology_of_gene_regulatory_elements/projects/miRDeep/index.html	[69]
miRTools	1.1	Detect novel miRNA based on HTS	http://centre.bioinformatics.zj.cn/mirtools/	[70]
starBase	1.3	Decoding microRNA-target and protein-RNA interaction	http://starbase.sysu.edu.cn/	[71]
IPA	1.4	Comprehensive software on biological analysis. Support miRNA target filtering	http://www.ingenuity.com/products/training.html	

HTS technology provides high performance and low-cost in detecting miRNA sequencing and therefore replaces traditional Sanger sequencing. The most famous HTS technologies are 454 sequencing [79] from Roche, Solexa sequencing [80] from Illumina, SOLiD sequencing [81] from ABI and SMRT [82] from Pacific Biosciences. Currently, there are several platforms based on HTS, established for scientific use, such as High-Throughput Genome Sequencing from the Gene Bank and the Princeton University High Throughput Sequencing Database. Specific to miRNAs, deep sequencing tools have been developed to analyse HTS results and then to detect novel miRNAs or extract miRNA expression profiles, for example, miRExpress [67], miRTools [70] and miRDeep [69].

Current experimental approaches to miRNA target identification mainly focus on the use of large scale mRNA expression profiling. The common way to uncover miRNA targets is to directly test miRNA expression levels on different mRNA profiling [83] or to use different phenotypes to test expression levels [84] based on microarray [85]. Various computational approaches [11,86-92] provide fast and low-cost support to experiments. Sequence complementarity, evolutionary conservation and free energy among miRNA-mRNA duplex are the most common features to identify miRNA targets. The most frequently used applications are TargetScan [61], PicTar [59], miRanda [64], RNAHybrid [60] etc. The combination of computational approaches with mRNA expression profiles have proved efficient in recent years, and it has been shown that they can effectively minimize the false positives of miRNA target prediction.

For miRNA target filtering, previous studies have shown that considering conservation in strains combined with performing seed matches [93,94] or evaluating the accessibility of binding sites [95] can facilitate miRNA target predictions. Target filtering approaches are then developed to post-process the results of prediction applications. miRTif [65], for example, filters the predicted targets by SVM scores to evaluate the accessibility of binding sites and the properties of miRNA-mRNA interactions, such as the value of seed complementarity and binding energy. Recent studies show that combining both conservation and accessibility [96] can achieve better results in filtering miRNA targets.

In PPI data filtering, computational approaches are widely used to improve the PPI data obtained from laboratory experiments. Some approaches check whether the interactions support genomic features such as sequence, structure and annotation information to check the possibility of real interactions [97]. Alternatively, computational approaches integrate several databases to obtain more reliable interaction data, for example, HitPredict [53] integrated the databases of IntAct, BIOGRID and HPRD, collecting 168,458 highly reliable interactions across nine species. iRefIndex [54] and SynechoNET [55] are integrated databases as well. For single databases, checking whether interacting proteins have homolog across one or more species has proved an effective way to obtain high quality interactions [98].

After the filtering procedure, we can obtain highly reliable miRNA-target and protein-protein interaction networks. For certain miRNAs, miRNA-regulated PPI sub-networks can be easily constructed by combining their target sites and PPI networks according to the requirement. The sub-network may include miRNAs, target proteins, proteins associated with target proteins and the associations between proteins.

### Features of miRNA-regulated PPI networks

When a miRNA-targeted protein network is available, the task is to analyse the features of the miRNA-regulated PPI network (Figure 2b). A miRNA-regulated PPI network is defined to identify the featured sub-networks of the miRNA-target protein network. Here, we firstly describe the PPI network features.

The features of PPI networks are defined with a focus on topological characteristics, such as connectivity, cluster coefficients, shortest path length and so on. PPI network features are commonly used to predict unannotated protein functions combined with Gene Ontology [99].

Connectivity or degree denotes the interacted number of a node. Proteins with high degree are called hub proteins [100]. Betweenness centrality is the proportion of the shortest path length passing through a vertex protein and all the shortest path lengths from one certain protein to another certain protein. The nodes which have higher betweenness centrality are called network bottlenecks. Close Centrality is then defined to measure the sum of the shortest paths between the target protein and other proteins [23,73]. (Local) Cluster coefficient measures the cluster tendency of a node in a network, which equals the ratio of all the real interaction numbers in a cluster and the number of possible connections. For a node *i*, Cluster Coefficient *C*_*i*_ *= 2n*_*i*_*/k (k-1)*, where *k* is the number of edges in node *i*, and n is the edge number for all *k* neighbours connecting to each other.

In miRNA-regulated PPI networks, miRNAs are classified by several properties: a) the miRNA family or homologous miRNAs, which denote the miRNA group whose conserved seed regions are common [61]; b) clustered miRNAs, which are miRNAs whose pairwise chromosomal distances are no more than 3000nt [101]; and c) miRNAs with common targets. miRNAs can directly or indirectly down-regulate 100–200 genes on average [102], and each gene can be targeted by multi-miRNAs. Therefore, it is possible for miRNAs to regulate common target genes. There are still other classification criteria such as gene expression level, target mRNA and protein stability and the impact on target mRNA degradation, which are also essential in further analysis.

The purpose of the basic study on miRNA-regulated PPI networks is to find new rules on miRNA regulation or protein interaction, based on constructed sub-networks between miRNAs and protein interaction networks. As described above, miRNAs and protein-protein interaction networks both have different features, and by combining the analysis of these features, it is possible to obtain useful information or rules on both miRNA regulation or protein interaction, such as the relationship between miRNAs and protein complexes, how miRNAs coordinate to regulate targets and so on.

### Current findings in the study of miRNA-regulated PPI networks

As listed in Table 2, we have divided the previous findings into three areas: correlation between protein connectivity and miRNA regulation complexity; miRNA-regulated specific proteins, the coordination role of miRNAs in regulating PPI networks; and identifying miRNA-regulated PPI networks in special diseases.

**Table 2:** Current findings in the study of miRNA-regulated PPI networks

**Research area**	**Description**
Correlation between protein connectivity and miRNA regulation complexity	A. There is positive correlation between miRNA target site types and its regulated protein connectivity. B. MiRNA target propensity may be due to high protein connectivity. C. MiRNA regulation propensity changes due to different hub proteins [72].
	miRNA targeted proteins have short distance and higher modularity than randomly selected proteins [73].
MiRNA-regulated specific proteins in PPI networks	A. MiRNAs that target a lower number genes have the propensity to regulate commonly expressed proteins rather than tissue-specific proteins. B. Commonly expressed proteins and tissue-specific proteins are always regulated together by a miRNA, and the numbers of protein expressed are close in both proteins [44].
The coordination role of MicroRNAs: miRNA clusters regulate PPI networks	miRNAs in the same clusters have the tendency to coordinate to regulate protein functions in protein-protein interaction networks [74].
The coordination role of MicroRNAs: miRNAs coordinate to regulate protein complex	A. MiRNAs coordinate to regulate protein complexes in posttranscriptional level. B. Correlations between the proteins exist in the same complex regulated by miRNAs [75].
The coordination role of MicroRNAs: miRNA crossingtalking with transcription factors	Crosstalk motifs between miRNAs and transcription factors motif demonstrate higher network properties in miRNA-regulated PPI networks [7]
Identifying miRNA-regulated PPI networks in special diseases	A. In gastric cancer [76]: six miRNA-regulated protein networks are identified in gastric cancer based on the human PPI network; it is suggested that miR-148a may resist tumor extension. B. In human ovarian cancer [77]: six miRNAs (hsa-mir-20a, hsa-mir-24-2, hsa-mir-34a, hsa-mir-21, hsamir-17 and hsa-mir-hsa-mir-155) and six TFs (BRCA1, SP1, ESR1, SMAD3, PO2F1 and TFE2) play key roles in ovarian cancer progression. C. In aging-related diseases [78]: 35 genes related to diseases associated with aging were identified.

#### Correlation between protein connectivity and miRNA regulation complexity

Liang and Li [72] found that multiple miRNA-regulated proteins have more interacting neighbours in a PPI network. They observed that the number of miRNA target site types increases with the increasing connectivity of a protein. Based on observations, they developed the prediction formula for the average number (y) of target site types in the 3′-UTR. If x is the range of mRNA-expressed tissues (0 < x < 73), y = 0.0225x + 1.774. Liang and Li subsequently revealed that miRNA propensity [103] to regulate certain biological processes may be due to the higher connectivity of proteins in the PPI network. Finally, they pointed out that regulation by miRNA plays a more important role in low clustering coefficient hub proteins (connectivity > 8) which are intended to coordinate several functions than those hub proteins with high clustering coefficients.

Hsu, Juan and Huang [73], who extended Liang and Li’s study, proposed that miRNA-regulated proteins are always the hub or bottleneck in the PPI network. To clarify this issue, they firstly defined four PPI features [23]: degree, cluster coefficient, betweenness centrality and closeness centrality. Two sub-networks were then defined referring to the relationships between miRNAs and the PPI network. In the experiment, the target genes were detected by TargetScan, and then the target proteins with the target genes regulated by certain miRNAs were collected as a sub-network L0, and all the interactions which contain target proteins were collected as sub-network L1. Z score and p value were then computed in sub-network L0 and L1. By comparing randomly selected protein sub-graphs, the paper suggested that miRNA target proteins have shorter distance and higher modularity than randomly selected proteins.

#### MiRNA-regulated specific proteins in PPI networks

Zhu *et al.*[44] explored the relationships between miRNA and tissue-specific proteins in the human PPI network. They selected 10 main human tissues and defined proteins which were expressed in less than 3 tissues as tissue-specific proteins, and proteins which were expressed in all 10 tissues were commonly expressed proteins. Based on the findings of [104], which determined that tissue-specific proteins have the propensity to interact with commonly expressed proteins, Zhu *et al*. further found that miRNAs which target a lower number of genes have the propensity to regulate commonly expressed proteins rather than tissue-specific proteins in the human PPI network. From observations of miRNA regulation in all 10 tissues, they pointed out that miRNA regulation maintained consistency, that is, commonly expressed proteins and tissue-specific proteins were always regulated together by a miRNA, and the numbers of protein expressed are close in both proteins.

#### The coordination role of MicroRNAs in regulating PPI networks

Firstly, clustered miRNAs may coordinate to regulate the PPI network. Yuan *et al*. [74] revealed that miRNAs in the same clusters coordinate to regulate protein functions in protein-protein interaction networks. 55 clusters were identified at the miRNA family level according to miRBase. The miRNAs in the same clusters were defined as sc-miRNAs. The coordination strength of sc-miRNAs is measured by the number of clusters which regulate the two interacting proteins and the coordination range is represented by the number of interactions which are regulated by the miRNAs in the same clusters. The measurement of coordination indicates that interacting proteins have a tendency to be regulated by sc-miRNAs. Furthermore, the authors also point out that the tendency is influenced by protein interaction features in the protein-protein interaction network, for example, distance (shortest path between proteins) and connectivity (protein neighbour numbers).

Secondly, miRNAs coordinate to regulate protein complex. Sass *et al.*[75] used protein complexes which are verified based on the PPI network to investigate the relationships between miRNAs and protein complexes at a system level. The significance of the associations between miRNAs and their target protein complexes were ranked based on the P-value resulting from Fisher’s exact test. According to the test, the complexes which were regulated by single miRNAs or miRNAs in the same clusters were ranked higher than the ones which were regulated by multi-miRNAs in different clusters. The paper also proved that there are correlations between proteins in the same complex which are regulated by miRNAs.

**Figure 3:**
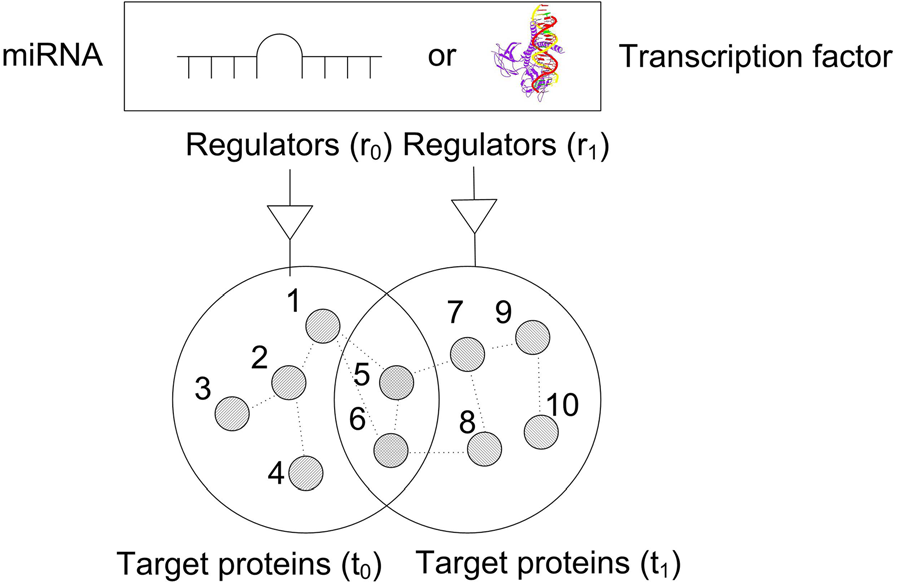
**Cooperating regulation motifs of miRNA and transcription factors: four regulator motifs: single regulation motif = {r**_**0**_**, 1,2,3,4,5,6} or {r**_**1**_**, 5,6,7,8,9,10}; co-regulation motif = {r**_**0**_**, r**_**1**_**, 5,6} crosstalk motif = {r**_**0**_**, r**_**1**_**,1,2,3,4,7,8,9,10}; If {5} or {6} does not exist, independent motif = {r**_**0**_**, 1,2,3,4,5,6} and {r**_**1**_**, 5,6,7,8,9,10}.**

Thirdly, miRNAs may coordinate with other regulators such as transcription factors (TFs). Based on previous findings, Cui *et al.*[39,105] found a correlation between transcription factors and miRNAs, and Lin *et al*. [7] extended this investigation to the human PPI network. Via the analysis of large gene/proteins of the regulators (TFs or miRNAs), the roles that regulators serve in the PPI network can be identified. Four types of motifs are defined in the paper for comparison purposes. These are single-regulation motifs, co-regulation motifs, crosstalk motifs and independent motifs. All these motifs contain both regulators and their target genes/proteins. As illustrated in Figure 3, for synergistic regulators r_0_ and r_1_, which have two common targets, and their targets t0 and t1, single regulation motif = {r_0_, t_0_} or {r_1_, t_1_} co-regulation motif = {r_0_, t_0_} ∩ {r_1_, t_1_}; crosstalk motif = {r_0_, t_0_} ∪ {r1, t_1_} − {r_0_, t_0_} ∩ {r1, t_1_}; if t_0_ and t_1_ do not have intersections, independent motif = {r_0_, t_0_} and {r_1_, t_1_}. PPI enrichment analysis and property analysis of the PPI network such as degree, closeness, density and so on are conducted to compare the significance of the four motifs in the PPI network. The results showed crosstalk motifs between miRNAs and TFs motifs, which demonstrated higher network properties such as higher degree, closeness, density etc., play a more important role in regulating the PPI network.

#### Identifying miRNA-regulated PPI networks in special diseases

Since the importance of miRNA-regulating PPI networks has been acknowledged, researchers have started to identify special miRNA-regulated networks in PPI networks related to disease. For example, Tseng [76] proposed an integrative method to identify miRNA-regulated protein networks specialized in gastric cancer. They combined miRNA targets and mRNA expression profiles and identified 16 miRNA-regulated protein networks in gastric cancer based on the human PPI network. Specially, they further investigated the network and functions of miR-148a which may resist tumor extension. Schmeier et al. [77] analysed miRNA-regulated PPI networks involving TFs in human ovarian cancer. They firstly generated miRNA-regulated networks based on 162 miRNAs and TFs related to ovarian cancer and human PPI data extracted from five databases, and then developed a node ranking algorithm in a weighted directed network to identify network hubs. Through the analysis of miRNAs and TFs linking to high ranking hubs, the authors determined some “key players” including miRNAs and TFs in ovarian cancer. Tacutu et al. [78,106] identified 35 genes related to aging related diseases (ARDs) from the miRNA-regulated PPI networks which they constructed from miRNA-regulated genes and the Common Gene Signature (in [78,107-109]; CGS denotes a PPI network based on the overlap between the PPI Human Longevity Network-http://www.netage-project.org[106] and human aging-related disease PPI networks).

## Conclusion

Through analysing miRNA-regulated PPI networks based on miRNA features and PPI network features, it can be seen that: a) proteins in PPI networks are different in miRNA regulation. miRNAs have the propensity to regulate higher connected proteins. Additionally, the target proteins have shorter and higher modularity than other random proteins. Especially for hub proteins, miRNAs seem to play more important roles in higher cluster coefficient proteins than lower ones. For proteins in special tissues, miRNAs that target a lower number of genes have the propensity to regulate commonly expressed proteins rather than tissue-specific proteins. Additionally, protein complexes tend to be regulated by miRNAs; b) miRNAs may cooperate to regulate target proteins with others. The collaborator can be other miRNAs in a cluster or it can also be TFs. Interacting proteins have the tendency to be regulated by miRNAs in the same cluster. On the other hand, the crosstalk between miRNAs and TFs plays a more important role in regulating proteins than other types, such as single-regulation, co-regulation and independent regulation. Finally, through analysing miRNA-regulated PPI networks in cancer, several key miRNAs associated with cancers can be identified. These findings suggest that by combining miRNAs and PPI network features, it is possible to obtain useful information or rules on both miRNA regulation and protein interaction.

Research on miRNA-regulated PPI networks is continuing and still has a long way to go. In one aspect, there are still other ways to further investigate miRNA regulation roles in the PPI network. For example, it is reported [99] that there is low connectivity among hub proteins in PPI networks, so is this a possible explanation for miRNA target propensity? We also notice that current studies on PPI networks are mostly focused on static PPI networks, however, PPIs may be expressed as a dynamic state to maintain stability in the external environment, hence, it would be interesting to study the relationship between miRNA-regulated dynamic PPI networks or even at a later stage, on data hub proteins. On the other hand, studies on miRNA-regulated networks can also be extended into another three aspects (if applicable): a) the improvement of current miRNA target prediction approaches by integrating protein-protein interaction data. Liang and Li [72] found a convincing relationship between miRNA target sites and PPI networks and then mentioned that integrating PPI networks may result in the improvement of miRNA target prediction; b) the improvement of the detection of signalling pathways by integrating miRNA target data. miRNA regulating pathways related to cancer have been the subject of a great deal of recent research. Conversely, it is also possible to use this regulatory mechanism to infer signal pathways, and hence provide theoretical support to current miRNA-regulated signalling transduction pathway research; c) the improvement of protein function prediction approaches by integrating protein-protein interaction data and miRNA target data. Current protein function prediction algorithms mostly rely on designed mathematical algorithms and single resources (e.g. only using the PPI network database), which results in lower applicability. miRNAs play a fine-tuning role in gene expression, the integrating miRNA-regulated data can result in the current protein function prediction to be more biologically meaningful, and in turn, result in the improvement of accuracy.

### Key points

•We divide current miRNA-regulated PPI networks into: 1) basic studies of miRNA-regulated PPI networks; and 2) identification of miRNA-regulated networks in diseases which covers most parts of current miRNA-regulated PPI network studies.

•We provide relatively complete resources to construct a miRNA-target protein network.

•We introduce the features of miRNA-regulated PPI networks and based on this, we provide a brief explanation on the methodologies of several studies on miRNA-regulated PPI networks.

•We review previous findings and propose several potential computational research directions which may be further explored in the future.

## Competing interests

The authors declare no competing financial interests.

## Authors’ contributions

WZ conceived and designed the study, YPP surprised the work. WZ and YPP wrote reviewed and edited the paper. All authors read and approved the final manuscript.

## Authors’ information

Wei Zhu is a Ph.D candidate in the Department of Computer Science and Computer Engineering at La Trobe University. The focus of his thesis is knowledge discovery in protein-protein interactions.

Yi-Ping Phoebe Chen is the Professor and Chair & Director of Research in the Department of Computer Science and Computer Engineering, La Trobe University, Melbourne, Australia. She is currently working on knowledge discovery technologies and is especially interested in their application to genomics and biomedical science. Her research focus is to find best solutions for mining, integrating and analysing complex data structures and functions for scientific and biomedical applications.

## References

[B1] Rodriguez A Griffiths-Jones S Ashurst JL Bradley A Identification of mammalian microRNA host genes and transcription units Genome research 2004 14 10A 1902 1910 10.1101/gr.2722704 15364901 PMC524413

[B2] Weber MJ New human and mouse microRNA genes found by homology search FEBS Journal 2005 272 1 59 73 15634332 10.1111/j.1432-1033.2004.04389.x

[B3] Lee RC Feinbaum RL Ambros V The C. elegans heterochronic gene lin-4 encodes small RNAs with antisense complementarity to lin-14 Cell 1993 75 5 843 854 10.1016/0092-8674(93)90529-Y 8252621

[B4] Lee Y Jeon K Lee JT Kim S Kim VN MicroRNA maturation: stepwise processing and subcellular localization The EMBO journal 2002 21 17 4663 4670 10.1093/emboj/cdf476 12198168 PMC126204

[B5] Chen F Chen YP Exploring cross-species-related miRNAs based on sequence and secondary structure IEEE transactions on bio-medical engineering 2010 57 7 1547 1553 20199930 10.1109/TBME.2010.2043734

[B6] An J Choi KP Wells CA Chen YP Identifying co-regulating microRNA groups Journal of bioinformatics and computational biology 2010 8 1 99 115 10.1142/S0219720010004574 20183876

[B7] Lin CC Chen YJ Chen CY Oyang YJ Juan HF Huang HC Crosstalk between transcription factors and microRNAs in human protein interaction network BMC systems biology 2012 6 18 10.1186/1752-0509-6-18 22413876 PMC3337275

[B8] Lu J Getz G Miska EA Alvarez-Saavedra E Lamb J Peck D Sweet-Cordero A Ebert BL Mak RH Ferrando AA MicroRNA expression profiles classify human cancers Nature 2005 435 7043 834 838 10.1038/nature03702 15944708

[B9] Tsuchiya S Okuno Y Tsujimoto G MicroRNA: biogenetic and functional mechanisms and involvements in cell differentiation and cancer Journal of pharmacological sciences 2006 101 4 267 270 10.1254/jphs.CPJ06013X 16921236

[B10] Tzur G Israel A Levy A Benjamin H Meiri E Shufaro Y Meir K Khvalevsky E Spector Y Rojansky N Comprehensive gene and microRNA expression profiling reveals a role for microRNAs in human liver development PloS one 2009 4 10 e7511 10.1371/journal.pone.0007511 19841744 PMC2760133

[B11] Maziere P Enright AJ Prediction of microRNA targets Drug discovery today 2007 12 11–12 452 458 17532529 10.1016/j.drudis.2007.04.002

[B12] Nagel R le Sage C Diosdado B van der Waal M Oude Vrielink JA Bolijn A Meijer GA Agami R Regulation of the adenomatous polyposis coli gene by the miR-135 family in colorectal cancer Cancer research 2008 68 14 5795 5802 10.1158/0008-5472.CAN-08-0951 18632633

[B13] Pasquinelli AE Reinhart BJ Slack F Martindale MQ Kuroda MI Maller B Hayward DC Ball EE Degnan B Muller P Conservation of the sequence and temporal expression of let-7 heterochronic regulatory RNA Nature 2000 408 6808 86 89 10.1038/35040556 11081512

[B14] Guo L Lu ZH The fate of miRNA* strand through evolutionary analysis: implication for degradation as merely carrier strand or potential regulatory molecule? PLoS One 2010 5 6 e11387 10.1371/journal.pone.0011387 20613982 PMC2894941

[B15] Cai Y Yu X Hu S Yu J A brief review on the mechanisms of miRNA regulation Genomics, proteomics & bioinformatics 2009 7 4 147 154 10.1016/S1672-0229(08)60044-3 20172487 PMC5054406

[B16] Yu B Qian T Wang Y Zhou S Ding G Ding F Gu X miR-182 inhibits Schwann cell proliferation and migration by targeting FGF9 and NTM, respectively at an early stage following sciatic nerve injury Nucleic Acids Res 2012 40 20 10356 10365 10.1093/nar/gks750 22917588 PMC3488220

[B17] Cheng C Bhardwaj N Gerstein M The relationship between the evolution of microRNA targets and the length of their UTRs BMC Genomics 2009 10 431 10.1186/1471-2164-10-431 PMC2758905 19751524

[B18] Ørom UA Nielsen FC Lund AH MicroRNA-10a binds the 5′UTR of ribosomal protein mRNAs and enhances their translation Molecular Cell 2008 30 460 471 10.1016/j.molcel.2008.05.001 18498749

[B19] Forman JJ Coller HA The code within the code MicroRNAs target coding regions Cell Cycle 2010 9 8 1533 1541 10.4161/cc.9.8.11202 20372064 PMC2936675

[B20] Rhoades MW Reinhart BJ Lim LP Burge CB Bartel B Bartel DP Prediction of plant microRNA targets Cell 2002 110 4 513 520 10.1016/S0092-8674(02)00863-2 12202040

[B21] Brennecke J Stark A Russell RB Cohen SM Principles of microRNA-target recognition PLoS biology 2005 3 3 e85 10.1371/journal.pbio.0030085 15723116 PMC1043860

[B22] Hartwell LH Hopfield JJ Leibler S Murray AW From molecular to modular cell biology Nature 1999 402 6761 Suppl C47 52 10591225 10.1038/35011540

[B23] Zhang SH Jin GX Zhang XS Chen LN Discovering functions and revealing mechanisms at molecular level from biological networks Proteomics 2007 7 16 2856 2869 10.1002/pmic.200700095 17703505

[B24] Fields S Song O A novel genetic system to detect protein-protein interactions Nature 1989 340 6230 245 246 10.1038/340245a0 2547163

[B25] Schwikowski B Uetz P Fields S A network of protein-protein interactions in yeast Nature biotechnology 2000 18 12 1257 1261 10.1038/82360 11101803

[B26] Stelzl U Worm U Lalowski M Haenig C Brembeck FH Goehler H Stroedicke M Zenkner M Schoenherr A Koeppen S A human protein-protein interaction network: a resource for annotating the proteome Cell 2005 122 6 957 968 10.1016/j.cell.2005.08.029 16169070

[B27] Ito T Chiba T Ozawa R Yoshida M Hattori M Sakaki Y A comprehensive two-hybrid analysis to explore the yeast protein interactome Proceedings of the National Academy of Sciences of the United States of America 2001 98 8 4569 4574 10.1073/pnas.061034498 11283351 PMC31875

[B28] Aebersold R Mann M Mass spectrometry-based proteomics Nature 2003 422 6928 198 207 10.1038/nature01511 12634793

[B29] Fields S High-throughput two-hybrid analysis: the promise and the peril Febs J 2005 272 21 5391 5399 10.1111/j.1742-4658.2005.04973.x 16262681

[B30] Zhu W Hou J Chen YP Exploiting multi-layered information to iteratively predict protein functions Mathematical biosciences 2012 236 2 108 116 10.1016/j.mbs.2012.02.004 22391459

[B31] Rogler CE Levoci L Ader T Massimi A Tchaikovskaya T Norel R Rogler LE MicroRNA-23b cluster microRNAs regulate transforming growth factor-β/bone morphogenetic protein signaling and liver stem cell differentiation by targeting Smads Hepatology 2009 50 2 570 584 10.1002/hep.22982 19582816

[B32] Mei J Bachoo R Zhang CL MicroRNA-146a inhibits glioma development by targeting Notch1 Mol Cell Biol 2011 31 17 3584 3592 10.1128/MCB.05821-11 21730286 PMC3165557

[B33] Papagiannakopoulos T Friedmann-Morvinski D Neveu P Dugas JC Gill RM Huillard E Liu C Zong H Rowitch DH Barres BA Pro-neural miR-128 is a glioma tumor suppressor that targets mitogenic kinases Oncogene 2012 31 15 1884 1895 10.1038/onc.2011.380 21874051 PMC4160048

[B34] Enright AJ Van Dongen S Ouzounis CA An efficient algorithm for large-scale detection of protein families Nucleic acids research 2002 30 7 1575 1584 10.1093/nar/30.7.1575 11917018 PMC101833

[B35] King AD Przulj N Jurisica I Protein complex prediction via cost-based clustering Bioinformatics 2004 20 17 3013 3020 10.1093/bioinformatics/bth351 15180928

[B36] Cho YR Hwang W Ramanathan M Zhang A Semantic integration to identify overlapping functional modules in protein interaction networks BMC bioinformatics 2007 8 265 10.1186/1471-2105-8-265 17650343 PMC1971074

[B37] Liu G Wong L Chua HN Complex discovery from weighted PPI networks Bioinformatics 2009 25 15 1891 1897 10.1093/bioinformatics/btp311 19435747

[B38] Inui M Martello G Piccolo S MicroRNA control of signal transduction Nat Rev Mol Cell Biol 2010 11 4 252 263 20216554 10.1038/nrm2868

[B39] Cui Q Yu Z Purisima EO Wang E Principles of microRNA regulation of a human cellular signaling network Molecular systems biology 2006 2 46 16969338 10.1038/msb4100089 PMC1681519

[B40] Carthew RW Sontheimer EJ Origins and mechanisms of miRNAs and siRNAs Cell 2009 136 4 642 655 10.1016/j.cell.2009.01.035 19239886 PMC2675692

[B41] Kim VN Han J Siomi MC Biogenesis of small RNAs in animals Nat Rev Mol Cell Biol 2009 10 2 126 139 10.1038/nrm2632 19165215

[B42] Selbach M Schwanhausser B Thierfelder N Fang Z Khanin R Rajewsky N Widespread changes in protein synthesis induced by microRNAs Nature 2008 455 7209 58 63 10.1038/nature07228 18668040

[B43] Baek D Villen J Shin C Camargo FD Gygi SP Bartel DP The impact of microRNAs on protein output Nature 2008 455 7209 64 71 10.1038/nature07242 18668037 PMC2745094

[B44] Zhu W Yang L Du Z MicroRNA regulation and tissue-specific protein interaction network PloS one 2011 6 9 e25394 10.1371/journal.pone.0025394 21980443 PMC3181334

[B45] Han JD Bertin N Hao T Goldberg DS Berriz GF Zhang LV Dupuy D Walhout AJ Cusick ME Roth FP Evidence for dynamically organized modularity in the yeast protein-protein interaction network Nature 2004 430 6995 88 93 10.1038/nature02555 15190252

[B46] Stark C Breitkreutz BJ Reguly T Boucher L Breitkreutz A Tyers M BioGRID: a general repository for interaction datasets Nucleic Acids Res 2006 34 Database issue D535 539 16381927 10.1093/nar/gkj109 PMC1347471

[B47] Shannon P Markiel A Ozier O Baliga NS Wang JT Ramage D Amin N Schwikowski B Ideker T Cytoscape: a software environment for integrated models of biomolecular interaction networks Genome Res 2003 13 11 2498 2504 10.1101/gr.1239303 14597658 PMC403769

[B48] Xenarios I Salwinski L Duan XJ Higney P Kim SM Eisenberg D DIP, the database of interacting proteins: a research tool for studying cellular networks of protein interactions Nucleic Acids Res 2002 30 1 303 305 10.1093/nar/30.1.303 11752321 PMC99070

[B49] Lu M Zhang Q Deng M Miao J Guo Y Gao W Cui Q An analysis of human microRNA and disease associations PLoS One 2008 3 10 e3420 10.1371/journal.pone.0003420 18923704 PMC2559869

[B50] Heikkinen L Kolehmainen M Wong G Prediction of microRNA targets in caenorhabditis elegans using a self-organizing map Bioinformatics 27 9 1247 1254 21422073 10.1093/bioinformatics/btr144

[B51] Pagel P Kovac S Oesterheld M Brauner B Dunger-Kaltenbach I Frishman G Montrone C Mark P Stumpflen V Mewes HW The MIPS mammalian protein-protein interaction database Bioinformatics 2005 21 6 832 834 10.1093/bioinformatics/bti115 15531608

[B52] Wu J Vallenius T Ovaska K Westermarck J Makela TP Hautaniemi S Integrated network analysis platform for protein-protein interactions Nat Methods 2009 6 1 75 77 10.1038/nmeth.1282 19079255

[B53] Patil A Nakai K Nakamura H HitPredict: a database of quality assessed protein-protein interactions in nine species Nucleic Acids Res 2011 39 Database issue D744 749 20947562 10.1093/nar/gkq897 PMC3013773

[B54] Razick S Magklaras G Donaldson IM iRefIndex: a consolidated protein interaction database with provenance BMC Bioinformatics 2008 9 405 10.1186/1471-2105-9-405 18823568 PMC2573892

[B55] Kim WY Kang S Kim BC Oh J Cho S Bhak J Choi JS SynechoNET: integrated protein-protein interaction database of a model cyanobacterium Synechocystis sp. PCC 6803 BMC Bioinformatics 2008 9 1 S20 10.1186/1471-2105-9-20 18315852 PMC2259421

[B56] Zhang Z Yu J Li D Liu F Zhou X Wang T Ling Y Su Z PMRD: plant microRNA database Nucleic Acids Res 2010 38 Database issue D806 813 19808935 10.1093/nar/gkp818 PMC2808885

[B57] Ashburner M Ball CA Blake JA Botstein D Butler H Cherry JM Davis AP Dolinski K Dwight SS Eppig JT Gene ontology: tool for the unification of biology: the gene ontology consortium Nat Genet 2000 25 1 25 29 10.1038/75556 10802651 PMC3037419

[B58] Hsu SD Lin FM Wu WY Liang C Huang WC Chan WL Tsai WT Chen GZ Lee CJ Chiu CM miRTarBase: a database curates experimentally validated microRNA-target interactions Nucleic acids research 2011 39 Database issue D163 169 21071411 10.1093/nar/gkq1107 PMC3013699

[B59] Krek A Grun D Poy MN Wolf R Rosenberg L Epstein EJ MacMenamin P da Piedade I Gunsalus KC Stoffel M Combinatorial microRNA target predictions Nat Genet 2005 37 5 495 500 10.1038/ng1536 15806104

[B60] Kruger J Rehmsmeier M RNAhybrid: microRNA target prediction easy, fast and flexible Nucleic Acids Res 2006 34 Web Server issue W451 454 16845047 10.1093/nar/gkl243 PMC1538877

[B61] Lewis BP Burge CB Bartel DP Conserved seed pairing, often flanked by adenosines, indicates that thousands of human genes are microRNA targets Cell 2005 120 1 15 20 10.1016/j.cell.2004.12.035 15652477

[B62] Antonov AV Dietmann S Wong P Lutter D Mewes HW GeneSet2miRNA: finding the signature of cooperative miRNA activities in the gene lists Nucleic Acids Research 2009 37 W323 W328 10.1093/nar/gkp313 19420064 PMC2703952

[B63] Nam S Li M Choi KM Balch C Kim S Nephew KP MicroRNA and mRNA integrated analysis (MMIA): a web tool for examining biological functions of microRNA expression Nucleic Acids Research 2009 37 W356 W362 10.1093/nar/gkp294 19420067 PMC2703907

[B64] Enright AJ John B Gaul U Tuschl T Sander C Marks DS MicroRNA targets in Drosophila Genome Biol 2003 5 1 R1 10.1186/gb-2003-5-1-r1 14709173 PMC395733

[B65] Yang Y Wang YP Li KB MiRTif: a support vector machine-based microRNA target interaction filter BMC Bioinformatics 2008 9 12 S4 19091027 10.1186/1471-2105-9-S12-S4 PMC2638144

[B66] Kozomara A Griffiths-Jones S miRBase: integrating microRNA annotation and deep-sequencing data Nucleic Acids Res 2010 39 Database issue D152 157 21037258 10.1093/nar/gkq1027 PMC3013655

[B67] Wang WC Lin FM Chang WC Lin KY Huang HD Lin NS miRExpress: analyzing high-throughput sequencing data for profiling microRNA expression BMC Bioinformatics 2009 10 328 10.1186/1471-2105-10-328 19821977 PMC2767369

[B68] Vergoulis T Vlachos IS Alexiou P Georgakilas G Maragkakis M Reczko M Gerangelos S Koziris N Dalamagas T Hatzigeorgiou AG TarBase 6.0: capturing the exponential growth of miRNA targets with experimental support Nucleic Acids Res 2012 40 Database issue D222 229 22135297 10.1093/nar/gkr1161 PMC3245116

[B69] Friedlander MR Chen W Adamidi C Maaskola J Einspanier R Knespel S Rajewsky N Discovering microRNAs from deep sequencing data using miRDeep Nature Biotechnology 2008 26 4 407 415 10.1038/nbt1394 18392026

[B70] Zhu E Zhao F Xu G Hou H Zhou L Li X Sun Z Wu J mirTools: microRNA profiling and discovery based on high-throughput sequencing Nucleic Acids Res 2010 38 Web Server issue W392 397 20478827 10.1093/nar/gkq393 PMC2896132

[B71] Yang JH Li JH Shao P Zhou H Chen YQ Qu LH starBase: a database for exploring microRNA-mRNA interaction maps from Argonaute CLIP-Seq and Degradome-Seq data Nucleic Acids Res 2011 39 Database issue D202 209 21037263 10.1093/nar/gkq1056 PMC3013664

[B72] Liang H Li WH MicroRNA regulation of human protein protein interaction network RNA 2007 13 9 1402 1408 10.1261/rna.634607 17652130 PMC1950750

[B73] Hsu CW Juan HF Huang HC Characterization of microRNA-regulated protein-protein interaction network Proteomics 2008 8 10 1975 1979 10.1002/pmic.200701004 18491312

[B74] Yuan X Liu C Yang P He S Liao Q Kang S Zhao Y Clustered microRNAs’ coordination in regulating protein-protein interaction network BMC systems biology 2009 3 65 10.1186/1752-0509-3-65 19558649 PMC2714305

[B75] Sass S Dietmann S Burk UC Brabletz S Lutter D Kowarsch A Mayer KF Brabletz T Ruepp A Theis FJ MicroRNAs coordinately regulate protein complexes BMC systems biology 2011 5 136 10.1186/1752-0509-5-136 21867514 PMC3170341

[B76] Tseng CW Lin CC Chen CN Huang HC Juan HF Integrative network analysis reveals active microRNAs and their functions in gastric cancer BMC systems biology 2011 5 99 10.1186/1752-0509-5-99 21703006 PMC3142228

[B77] Schmeier S Schaefer U Essack M Bajic VB Network analysis of microRNAs and their regulation in human ovarian cancer BMC systems biology 2011 5 183 10.1186/1752-0509-5-183 22050994 PMC3219655

[B78] Tacutu R Budovsky A Wolfson M Fraifeld VE MicroRNA-regulated protein-protein interaction networks: how could they help in searching for pro-longevity targets? Rejuvenation research 2010 13 2–3 373 377 20367577 10.1089/rej.2009.0980

[B79] Margulies M Egholm M Altman WE Attiya S Bader JS Bemben LA Berka J Braverman MS Chen YJ Chen Z Genome sequencing in microfabricated high-density picolitre reactors Nature 2005 437 7057 376 380 16056220 10.1038/nature03959 PMC1464427

[B80] Cokus SJ Feng S Zhang X Chen Z Merriman B Haudenschild CD Pradhan S Nelson SF Pellegrini M Jacobsen SE Shotgun bisulphite sequencing of the Arabidopsis genome reveals DNA methylation patterning Nature 2008 452 7184 215 219 10.1038/nature06745 18278030 PMC2377394

[B81] Cloonan N Forrest AR Kolle G Gardiner BB Faulkner GJ Brown MK Taylor DF Steptoe AL Wani S Bethel G Stem cell transcriptome profiling via massive-scale mRNA sequencing Nat Methods 2008 5 7 613 619 10.1038/nmeth.1223 18516046

[B82] Flusberg BA Webster DR Lee JH Travers KJ Olivares EC Clark TA Korlach J Turner SW Direct detection of DNA methylation during single-molecule, real-time sequencing Nat Methods 2010 7 6 461 465 10.1038/nmeth.1459 20453866 PMC2879396

[B83] Herrera BM Lockstone HE Taylor JM Wills QF Kaisaki PJ Barrett A Camps C Fernandez C Ragoussis J Gauguier D MicroRNA-125a is over-expressed in insulin target tissues in a spontaneous rat model of Type 2 Diabetes Bmc Medical Genomics 2009 2 10.1186/1755-8794-2-54 PMC2754496 19689793

[B84] Ambs S Prueitt RL Yi M Hudson RS Howe TM Petrocca F Wallace TA Liu CG Volinia S Calin GA Genomic profiling of microRNA and messenger RNA reveals deregulated microRNA expression in prostate cancer Cancer Res 2008 68 15 6162 6170 10.1158/0008-5472.CAN-08-0144 18676839 PMC2597340

[B85] Dai Y Zhou X Computational methods for the identification of microRNA targets Open Access Bioinformatics 2010 2 29 39 22162940 10.2147/OAB.S6902 PMC3233190

[B86] Zhang BH Pan XP Wang QL Cobb GP Anderson TA Computational identification of microRNAs and their targets Computational Biology and Chemistry 2006 30 6 395 407 10.1016/j.compbiolchem.2006.08.006 17123865

[B87] Min H Yoon S Got target?: computational methods for microRNA target prediction and their extension Experimental and Molecular Medicine 2010 42 4 233 244 10.3858/emm.2010.42.4.032 20177143 PMC2859323

[B88] Chaudhuri K Chatterjee R MicroRNA detection and target prediction: integration of computational and experimental approaches DNA and Cell Biology 2007 26 5 321 337 10.1089/dna.2006.0549 17504028

[B89] Dai X Zhuang Z Zhao PX Computational analysis of miRNA targets in plants: current status and challenges Brief Bioinform 2010 12 2 115 121 20858738 10.1093/bib/bbq065

[B90] Wang X Computational prediction of microRNA targets Methods Mol Biol 2010 667 283 295 10.1007/978-1-60761-811-9_19 20827541

[B91] Hammell M Computational methods to identify miRNA targets Seminars in cell & developmental biology 2010 21 7 738 744 10.1016/j.semcdb.2010.01.004 20079866 PMC2891825

[B92] Garzon R Marcucci G Croce CM Targeting microRNAs in cancer: rationale, strategies and challenges Nature reviews Drug discovery 2010 9 10 775 789 10.1038/nrd3179 20885409 PMC3904431

[B93] Maragkakis M Alexiou P Papadopoulos GL Reczko M Dalamagas T Giannopoulos G Goumas G Koukis E Kourtis K Simossis VA Accurate microRNA target prediction correlates with protein repression levels BMC Bioinformatics 2009 10 295 10.1186/1471-2105-10-295 19765283 PMC2752464

[B94] Murphy E Vanicek J Robins H Shenk T Levine AJ Suppression of immediate-early viral gene expression by herpesvirus-coded microRNAs: implications for latency Proc Natl Acad Sci USA 2008 105 14 5453 5458 10.1073/pnas.0711910105 18378902 PMC2291141

[B95] Kertesz M Iovino N Unnerstall U Gaul U Segal E The role of site accessibility in microRNA target recognition Nat Genet 2007 39 10 1278 1284 10.1038/ng2135 17893677

[B96] Marin RM Vanicek J Optimal use of conservation and accessibility filters in MicroRNA target prediction PLoS One 2012 7 2 10.1371/journal.pone.0032208 PMC3288066 22384176

[B97] Patil A Nakamura H Filtering high-throughput protein-protein interaction data using a combination of genomic features BMC bioinformatics 2005 6 100 10.1186/1471-2105-6-100 15833142 PMC1127019

[B98] Patil A Nakamura H Filtering high-throughput protein-protein interaction data using a combination of genomic features BMC Bioinformatics 2005 6 6 10.1186/1471-2105-6-6 15833142 PMC1127019

[B99] Maslov S Sneppen K Specificity and stability in topology of protein networks Science 2002 296 5569 910 913 10.1126/science.1065103 11988575

[B100] Gursoy A Keskin O Nussinov R Topological properties of protein interaction networks from a structural perspective Biochemical Society transactions 2008 36 Pt 6 1398 1403 19021563 10.1042/BST0361398 PMC7243876

[B101] Altuvia Y Landgraf P Lithwick G Elefant N Pfeffer S Aravin A Brownstein MJ Tuschl T Margalit H Clustering and conservation patterns of human microRNAs Nucleic Acids Res 2005 33 8 2697 2706 10.1093/nar/gki567 15891114 PMC1110742

[B102] Lim LP Lau NC Garrett-Engele P Grimson A Schelter JM Castle J Bartel DP Linsley PS Johnson JM Microarray analysis shows that some microRNAs downregulate large numbers of target mRNAs Nature 2005 433 7027 769 773 10.1038/nature03315 15685193

[B103] Bartel DP MicroRNAs: genomics, biogenesis, mechanism, and function Cell 2004 116 2 281 297 10.1016/S0092-8674(04)00045-5 14744438

[B104] Bossi A Lehner B Tissue specificity and the human protein interaction network Molecular systems biology 2009 5 260 19357639 10.1038/msb.2009.17 PMC2683721

[B105] Cui Q Yu Z Pan Y Purisima EO Wang E MicroRNAs preferentially target the genes with high transcriptional regulation complexity Biochemical and biophysical research communications 2007 352 3 733 738 10.1016/j.bbrc.2006.11.080 17141185

[B106] Tacutu R Budovsky A Fraifeld VE The NetAge database: a compendium of networks for longevity, age-related diseases and associated processes Biogerontology 2010 11 4 513 522 10.1007/s10522-010-9265-8 20186480

[B107] Chen YPP Chen F Using bioinformatics techniques for gene identification in drug discovery and development Current Drug Metabolism 2008 9 6 567 573 10.2174/138920008784892056 18680477

[B108] Chen Q Chen YPP Mining frequent patterns for AMP-activated protein kinase regulation on skeletal muscle BMC Bioinformatics 2006 7 394 10.1186/1471-2105-7-394 16939655 PMC1574354

[B109] Song X Wang M Chen YPP Wang H Han P Sun H Prediction of pre-miRNA with multiple stem-loops using pruning algorithm Computers in Biology and Medicine 2013 43 5 409 416 10.1016/j.compbiomed.2013.02.003 23566387

